# TAD reorganization: a new mechanism for cell fate determination

**DOI:** 10.1186/s13619-021-00100-9

**Published:** 2021-12-02

**Authors:** Zhou Songyang

**Affiliations:** School of Life Sciences, Sun Yat-sen University, Guangzhou, P.R. China

Revealing how master transcription factors promote cell fate transitions through controlling TAD reorganization, in the recent issue of *Cell Stem Cell*, Wang et al. (2021) illustrate the phase separation of OCT4 controls TAD reorganization for cell fate transitions.

The eukaryotic three-dimensional (3D) genome is organized in a hierarchical order, mainly comprising compartments, topological-associated domains (TADs), and chromatin loops (Dixon et al., 2012; Lieberman-Aiden et al., 2009; Rao et al., 2014). 3D chromatin architectures are drastically altered during cell fate transitions, which plays an important role for these processes. Unlike compartments and chromatin loops, TADs are usually considered to be stable among different cell types and species (Dixon et al., 2012). However, recent studies have reported the loss of TADs during pluripotent stem cell (PSC) differentiation (Bonev et al., 2017; Zhang et al., 2019), indicating that TADs are likely to reorganize. Therefore, it is significant to clarify the role of TAD reorganization in regulating cell fate transitions, and the mechanism by which master transcription factors regulate TAD reorganization.

The authors started their journey by investigating whether TAD reorganization contributes to cell fate transitions. They employed two elegant methods to manipulate TAD reorganization. The first is chemical-dependent artificial looping. In this method, two genome loci belonging to adjacent TADs were targeted by dCas9-ABI or dCas9-PYL1, respectively. These two dCas9 proteins can be linked together with the small molecular chemical abscisic acid (ABA), and consequently looping of the targeted loci would be established upon ABA addition (Morgan et al., 2017). This looping may shorten the distance of two TADs, which may induce TAD merging. The second is boundary deletion via a CRISPR/Cas9 genome editing tool. For both methods, the two TADs were successfully merged as evidenced by Hi-C heatmap and increased insulation score. Importantly, TAD reorganization, induced by both methods, enhanced reprogramming efficiency, demonstrating that TAD reorganization contributes to cell fate transitions (Fig. 1A).

**Fig. 1:**
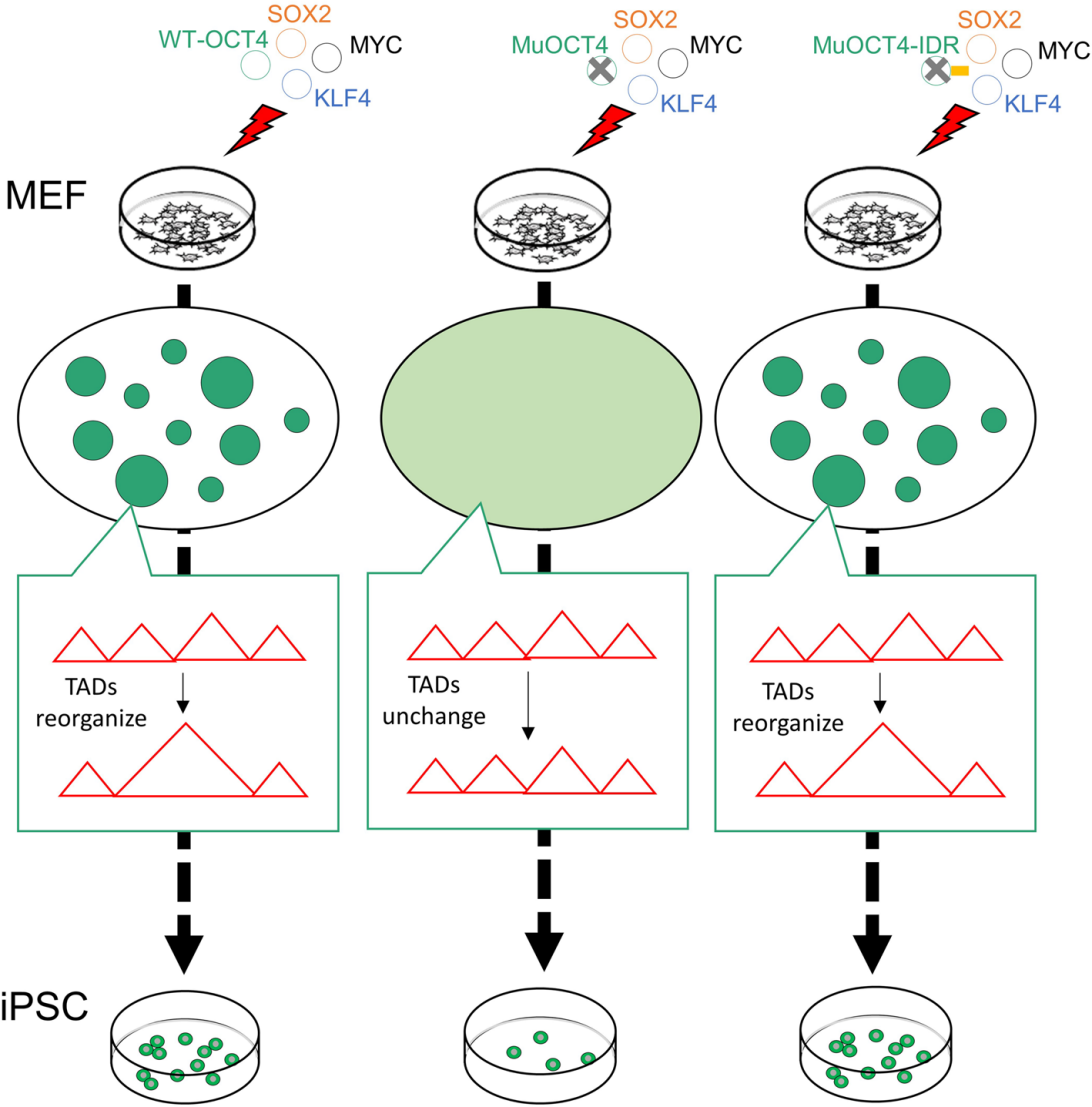
Phase Separation of OCT4 Controls TAD Reorganization for Cell Fate Transitions. (**A**) TAD reorganization can be manipulated by artificial looping or boundary deletion, respectively. Manipulation of TAD reorganization in MEFs can significantly enhance reprogramming efficiency. (**B**) OCT4 is phase separated during reprogramming. IDR mutation disrupts OCT4 phase separation, which can be rescued by fusing the IDR of FUS behind OCT4 mutant. OCT4 mutants attenuate TAD reorganization, which can be restored by IDR fusion. (**C**) OCT4 mutants reduce reprogramming efficiency, which can be recovered by IDR fusion

The authors then asked whether master transcription factor OCT4 directly regulates TAD reorganization. They performed OCT4 HiChIP to identify OCT4-mediated chromatin loops (OCT4 loops) during reprogramming, and found that the dynamic of OCT4 loops can induce TAD reorganization by regulating CTCF binding at TAD boundaries. Next, they performed protein immunoprecipitation followed by mass spectrometry (IP-MS) to identify the interacting proteins of OCT4. They found OCT4 interacts with the key structuring factors, such as CTCF, YY1 and cohesin proteins, and co-enriches with these factors at OCT4 loop anchors. When acute deletion of OCT4, the enrichment of the structuring factors at loop anchors is decreased and considerable chromatin loops disappear. These results demonstrate that OCT4 is required for structuring factors to form chromatin loops, which induces TAD reorganization by regulating CTCF binding at TAD boundaries. This suggests that OCT4 directly controls TAD reorganization.

The authors then investigated whether phase separation of master transcription factors promotes cell fate transitions. First, they showed that OCT4 forms phase-separated condensates with liquid-like behavior during reprogramming. Second, to evaluate the role of OCT4 phase separation in regulating reprogramming, they generated two OCT4 mutants predicted to disrupt OCT4 phase separation, one with acidic residues to alanine mutations in the intrinsically disordered regions (IDRs) (Boija et al., 2018), and the other with deleted IDR C-terminus (Fig. 1B). Both mutants can effectively disrupt OCT4 phase separation, which attenuates reprogramming. Third, to further verify the role of OCT4 phase separation in cell fate determination, the disabled phase-separated capacity of OCT4 mutants is rescued by fusing an IDR of FUS protein, which is a well-known region to drive liquid-like phase separation (Patel et al., 2015). Interestingly, rescue of OCT4 phase separation restores reprogramming (Fig. 1C), supporting the model that manipulation of phase separation can change cell fate transitions.

Finally, the authors asked the key question whether phase separation regulates TAD reorganization. To answer this question, the authors compared the effects of wildtype and various mutant forms of OCT4 on the alteration of TAD patterns during reprogramming. WT OCT4 induced significantly boundary change compare to negative controls, whereas OCT4 mutants only led to modest boundary change. This indicates that OCT4 can induce TAD reorganization in a phase separation-dependent manner. Importantly, the TAD patterns of FUS rescued-OCT4 mutants are similar to those of WT OCT4 but not IDR OCT4 mutants, demonstrating that rescue of OCT4 phase separation can restore TAD reorganization (Fig. 1B). These results for the first time to demonstrate the causal relationship between phase separation and higher-order 3D genome architectures.

Together, by employing the multiomics experiments and analyses including 3D genome, epigenome, proteome and transcriptome during somatic cell reprogramming, the authors discovered 1) the pattern that TADs are reorganized during cell fate transitions, 2) the mechanism by which master transcription factor OCT4 regulates TAD reorganization, and 3) how to guide cell fate transitions in the view of 3D genome. New methods were set up to control cell fate transitions by manipulating TAD structures or phase separation, and new algorithm was developed to precisely predict novel cell fate regulators. By establishing the causal relationship between phase separation and TAD reorganization for the first time, the authors provide new insights how master transcription factor controls cell fate transitions in the view of 3D genome.

This paper brought both theoretical and technical breakthroughs. In theory, it challenges the view that TADs are stable, and gives the role of TAD reorganization in gene expressions and cell fate determination. In technique, it provides the new methods how to artificially manipulate TAD structure or phase-separated ability of proteins. These methods are significant for further research in the fields of 3D genome and phase separation. Furthermore, new algorithm TADMAN was developed to precisely predict novel cell fate regulators. This algorithm can be used not only to identify normal cell fate transitions, but also to explore regulators in diseases such as cancer and premature aging.

## Data Availability

N/A

## References

[CR1] Boija A, Klein IA, Sabari BR, Dall'Agnese A, Coffey EL, Zamudio AV, Li CH, Shrinivas K, Manteiga JC, Hannett NM (2018). Transcription factors activate genes through the phase-separation capacity of their activation domains. Cell.

[CR2] Bonev B, Cohen NM, Szabo Q, Fritsch L, Papadopoulos GL, Lubling Y, Xu XL, Lv XD, Hugnot JP, Tanay A (2017). Multiscale 3D genome rewiring during mouse neural development. Cell.

[CR3] Dixon JR, Selvaraj S, Yue F, Kim A, Li Y, Shen Y, Hu M, Liu JS, Ren B (2012). Topological domains in mammalian genomes identified by analysis of chromatin interactions. Nature.

[CR4] Lieberman-Aiden E, van Berkum NL, Williams L, Imakaev M, Ragoczy T, Telling A, Amit I, Lajoie BR, Sabo PJ, Dorschner MO (2009). Comprehensive mapping of long-range interactions reveals folding principles of the human genome. Science.

[CR5] Morgan SL, Mariano NC, Bermudez A, Arruda NL, Wu F, Luo Y, Shankar G, Jia L, Chen H, Hu JF (2017). Manipulation of nuclear architecture through CRISPR-mediated chromosomal looping. Nat Commun.

[CR6] Patel A, Lee HO, Jawerth L, Maharana S, Jahnel M, Hein MY, Stoynov S, Mahamid J, Saha S, Franzmann TM (2015). A liquid-to-solid phase transition of the ALS protein FUS accelerated by disease mutation. Cell.

[CR7] Rao SS, Huntley MH, Durand NC, Stamenova EK, Bochkov ID, Robinson JT, Sanborn AL, Machol I, Omer AD, Lander ES (2014). A 3D map of the human genome at kilobase resolution reveals principles of chromatin looping. Cell.

[CR8] Wang J, Yu H, Ma Q, Zeng P, Wu D, Hou Y, et al. Phase separation of OCT4 controls TAD reorganization to promote cell fate transitions. Cell Stem Cell. 2021. 10.1016/j.stem.2021.04.023 34038708

[CR9] Zhang Y, Li T, Preissl S, Amaral ML, Grinstein JD, Farah EN, Destici E, Qiu Y, Hu R, Lee AY (2019). Transcriptionally active HERV-H retrotransposons demarcate topologically associating domains in human pluripotent stem cells. Nat Genet.

